# The antitumor activity of hPRDX5 against pancreatic cancer and the possible mechanisms

**DOI:** 10.1590/1414-431X2022e12324

**Published:** 2022-09-12

**Authors:** Lihua Cui, Yuanyuan Jin, Sen Zou, Jing Xun, Xiangyang Yu, Qi Zhang, Zhaoyong Yang

**Affiliations:** 1 NHC Key Laboratory of Biotechnology of Antibiotics, Institute of Medicinal Biotechnology, Chinese Academy of Medical Sciences, No. 1 Tiantanxili, Dongcheng District, Beijing, China; 2 Tianjin Key Laboratory of Acute Abdomen Disease Associated Organ Injury and ITCWM Repair, Tianjin Nankai Hospital, Tianjin, China; 3 Department of Gastrointestinal Surgery, Integrated Chinese and Western Medicine Hospital, Tianjin University, Tianjin, China

**Keywords:** Recombinant human peroxiredoxin-5, Pancreatic cancer, Immunotherapy, Immune cells activation, Myeloid-derived suppressor cells

## Abstract

Recombinant human peroxiredoxin-5 (hPRDX5), isolated from anti-cancer bioactive peptide (ACBPs), shows a homology of 89% with goat peroxiredoxin-5 (gPRDX5) and is reported to display anti-tumor activity *in vivo*. Herein, we explored the effect of hPRDX5 and the responsible mechanism in treating pancreatic cancer. Tumor-bearing mice were randomly divided into normal PBS group and treatment group (n=5; 10 mg/kg hPRDX5). Flow cytometry was employed to examine lymphocytes, myeloid-derived suppressor cell subsets, and the function proteins of natural killer (NK) cells in peripheral blood, spleen, and tumor tissues of mice. Western blot was used to measure the protein expressions of the key nodes in TLR4-MAPK-NF-κB signaling pathway. The rate of tumor suppression was 57.6% at a 10 mg/kg dose in orthotopic transplanted tumor mice. Moreover, the population of CD3+CD4+T cells, NK cells, and CD3+CD8+T cells was significantly increased in the tumor tissue of the hPRDX5 group, while the proportion of granulocytic-myeloid-derived suppressor cells decreased slightly. In addition, after treatment with hPRDX5, the percentage of NK cells in blood increased more than 4-fold. Our findings indicated that hPRDX5 effectively suppressed pancreatic cancer possibly via the TLR4-MAPK-NF-κB signaling cascade; hence hPRDX5 could be a prospective immunotherapy candidate for treating pancreatic cancer.

## Introduction

Pancreatic cancer is a serious disease of the digestive system. Unlike many other malignant tumors, the incidence and mortality of pancreatic cancer is growing, and it will be the second-leading cause of death-related to cancer by 2030 in the United States (1). At the time of diagnosis, around 50% of patients have developed metastasis, and the average survival time rarely exceeds 6 months (2). Currently, the routine treatment of advanced pancreatic cancer focuses mainly on alleviating pain and improving quality of life. This highlights the continued need for new and effective therapies for pancreatic cancer.

Immunotherapy is a promising approach for the treatment of many malignant tumors (3-6) but has not been applied to pancreatic cancer. Immune checkpoint inhibitors provided promising data at early stages but disappointing results at late stages (7,8). The most important reason for failures is the tumor immunosuppressive microenvironment (TME). In recent years, it has been found that pancreatic cancer cells escape host immune surveillance by mobilizing a variety of host immune cells to create a TME. Lymphocytes are the main effector cells of antitumor immune response. However, patients with pancreatic cancer show decreased numbers of circulating lymphocytes compared to healthy subjects. It is reported that natural killer (NK) cells in pancreatic cancer patients have the ability of lysing tumor cells (9), and high levels of NK cells in the tumor was associated with a better prognosis (10). However, even in the early stage of the disease, the lytic activity of NK cells is weak and becomes even weaker as the disease progresses (11,12). Myeloid-derived suppressor cells (MDSCs), identified as heterogeneous myeloid progenitor cells and immature myeloid cells, play an immunosuppressive role in cancer (13). MDSCs can suppress the function of T cells in several ways. Once activated, MDSCs can exert the activity of immunosuppressive cells by increasing the levels of arginase 1, nitric oxide, and nitric oxide synthase by monocytic (M)-MDSCs and high ROS production by granulocytic (G)-MDSCs (14-16). Therefore, targeting the restoration of the host's immunity can be an effective treatment strategy for patients with pancreatic cancer.

In the 1990s, a natural peptide mixture with relatively small molecular weight (16.7 KD) was isolated and extracted by researchers from goat spleen immunized with human gastric tumor protein extract. The mixture has excellent antitumor activity and no side effects, which is why it is named anti-cancer bioactive peptide (ACBPs). However, the complex procedure, time-consuming immunization of the goat, and the low output of peptide extraction from goat spleen hinder further study of its biological function and clinical application as an anticancer agent. In order to solve these problems, our group has carried out relevant research. First, we confirmed the sequence information of the important single component peptide with antitumor activity by 2D nano-LC-ESILTQ-Orbitrap MS/MS. On this basis, we determined that the target peptide was goat peroxiredoxin-5 (gPRDX5). The pure gPRDX5 was then obtained by heterogeneous expression, and the anticancer bioactivity was confirmed with several types of tumor-cells (17). To solve the potential safety problem of gPRDX5 applied to humans, we selected human peroxiredoxin-5 (hPRDX5), which has 89% similarity to gPRDX5 for further studies with the assistance of bioinformatics methods. The results showed that hPRDX5 had similar antitumor activities with gPRDX5, which was evaluated in mouse tumor models with colon cancer cell-C26 or melanoma cell-B16. Moreover, the results also suggested that hPRDX5 could counteract immunosuppression by promoting the development of immune organs, expanding lymphocyte population, and upregulating serum cytokines (18).

Currently, there are over 400 marketed recombinant products (peptides and proteins) and another 1300 are undergoing clinical trials. Oncology is one of the areas that dominate the biopharmaceutical market for therapeutic indications. In the context of the expanding protein drug market, there is a general consensus about the need to develop drugs that are stable *in vivo* and delivered in a cell- or tissue-targeted manner to reduce doses, production costs, and side effects. hPRDX5 is produced in *Escherichia coli*, which is usually the most preferred method due to its high degree of elaboration, rapid growth of biomass, and relatively low cost. However, in *E. coli*, most recombinant proteins are produced in insoluble form and therefore require additional efforts to accomplish recombinant protein refolding, which is difficult to scale and often reduces the yield of the target product. To determine the function and efficacy of hPRDX5, we will develop efficient bioprocessing strategies for hPRDX5 including various expression systems and development of a bioprocess to optimize its structure to increase stability of the product and prevent its unwanted degradation.

In this study, considering the immunity system of pancreatic cancer and the immune regulation function of hPRDX5, we explored the effect and mechanisms of hPRDX5 on pancreatic cancer in C57BL/6 mice by analyzing tumor growth, distribution of lymphocyte subsets, and function protein expressions, and by illustrating the mechanism of hPRDX5 in enhancing the cytotoxicity of NK cells *in vivo* and *in vitro*. Moreover, NK-92 cells were used to clarify the antitumor effect of hPRDX5 primarily through the TLR4-MAPK-NF-κB signaling pathways.

## Material and Methods

### Cell culture and treatment

The human NK-92 cell line and the mouse pancreatic cancer cell line Panc02 were purchased from Basic Medical Institute, Chinese Academy of Medical Sciences (China) and cultured according to the instructions. NK-92 cells were cultured in special alpha minimum essential medium (α-MEM), purchased from Procell (China). Panc02 cells were cultured in Dulbecco's modified Eagle's medium (DMEM; Gibco Chemical Co., USA) supplemented with 10% fetal bovine serum (FBS; Gibco Chemical Co.) and 1% penicillin/streptomycin (Gibco Chemical Co.). Cultures were maintained at 37°C in a humidified 5% CO_2_ incubator.

The chopped tumor tissue was digested by shaking with the prepared digestive solution. After grinding the tumor tissue, the tumor cell suspension was collected and the isolation solution of mononuclear cells from tumor infiltrating tissue of mice was added to obtain the tumor infiltrating mononuclear cells. The function protein expressions of IFN-γ, granzyme B, and perforin on NK cells were measured by flow cytometry.

NK cells were isolated from the spleen of healthy female C57BL/6 mice (Beijing Vital River Laboratory Animal Technology Co., Ltd. China) by magnetic bead sorting. The cells were cultured with hPRDX5 at different concentrations (5, 10, 50 μg/mL) for 24 h. Then, the function protein expressions of NK cells, IFN-γ, granzyme B, and perforin, were measured by flow cytometry.

### *In vivo* animal studies

All the animal experiments complied with the national regulations and management guidelines for experimental animal studies. The wild-type C57BL/6 female mice (Beijing Vital River Laboratory Animal Technology Co., Ltd.) were housed under standard conditions, with controlled temperature (20-25°C) and relative humidity (40-60%) in the specific pathogen-free laboratory animal room. Panc02 cells were collected and inoculated into the pancreas of mice. The tumor-bearing mice were randomly divided into treatment group (10 mg/kg) of hPRDX5 (n=5 mice per group) and control group (n=5, PBS). hPRDX5 was administered intraperitoneally for 14 days from the third day after surgery. At the end of the experiment, the peripheral blood was collected, and the mice were euthanized. Then, the tumor and spleen were excised, weighed, and photographed.

### Hematoxylin and eosin staining

Mouse pancreatic tumor tissues were separated from the pancreas and fixed with 4% paraformaldehyde, then dehydrated, and paraffin-embedded. Four micrometers-thick tissue sections were stained with H&E (Solarbio, China) according to the instructions. The sections were photographed by a Leica microscope (Germany).

### Flow cytometry

Mononuclear cells were isolated from spleen, blood, and tumor tissues of mice. The cells were stained with the monoclonal antibodies (Biolegend, USA) of (FITC)-conjugated anti-CD3, (PE)-conjugated anti-CD4, (APC)-conjugated anti-CD8, (PE)-conjugated anti-NK1.1, (FITC)-conjugated anti-CD11b, (PE)-conjugated anti-Ly6C, and (APC)-conjugated anti-Ly6G for the analysis of percentage of T cells, NK cells, and MDSC cells. The cells were stained with the monoclonal antibodies of (PerCP-Cy5.5)-conjugated anti-NK1.1, (FITC)-conjugated anti-IFN-γ, (PerCP-Cy7)-conjugated anti-granzyme B, and (PE)-conjugated anti-perforin for the expression analysis of function proteins in NK cells. All stained cells were incubated in the dark for 15 min, followed by washing with PBS 3 times, re-suspended with 200 μL PBS, and analyzed by a flow cytometer.

### Western blot

For the western blot experiment, NK-92 cells were treated with or without hPRDX5 (5, 10, and 50 μg/mL) for 24 h. In some experiments, NK92 cells were pre-treated with TLR4 inhibitor resatorvid (Selleck Chemicals, USA) for 1 h, and then hPRDX5 (50 μg/mL) was added for 24 h. The cells were collected and lysed in RIPA buffer containing PMFS protease inhibitor. The protein concentration was determined by a BCA protein assay. Then, the proteins were separated by SDS-PAGE gels and transferred to a PVDF membrane. The PVDF membrane was incubated with 5% bovine serum albumin for 1.5 h at room temperature, and the primary antibodies (Abcam, USA) anti-JNK, anti-ERK, anti-p38, anti-NF-κB, anti-p-JNK, anti-p-ERK, anti-p-p38, anti-p-NF-κB, and anti-GAPDH were incubated separately overnight at 4°C. Finally, secondary antibodies labeled with HRP were incubated at room temperature for 2 h. The protein blots were formed with the Beyo ECL Plus reagent (Beyotime Biotechnology, China), and the images were obtained in a ChemiDoc MP Imaging System (Bio-Rad, USA).

### Statistical analysis

All data are reported as means±SD. Analyses were performed with GraphPad Prism version 7.00 for Windows (USA). For the statistical analysis of normally distributed data sets, a Student's *t*-test was used to compare two unpaired treatment groups or a one-way ANOVA followed by Tukey's multiple comparisons test was used for multiple comparisons. The survival difference between groups was calculated with a log-rank test. When the P-value was less than 0.05, the difference was considered significant.

## Results

### hPRDX5 exhibited *in vivo* antitumor activity in mouse orthotopic transplantation model

**Figure 1:**
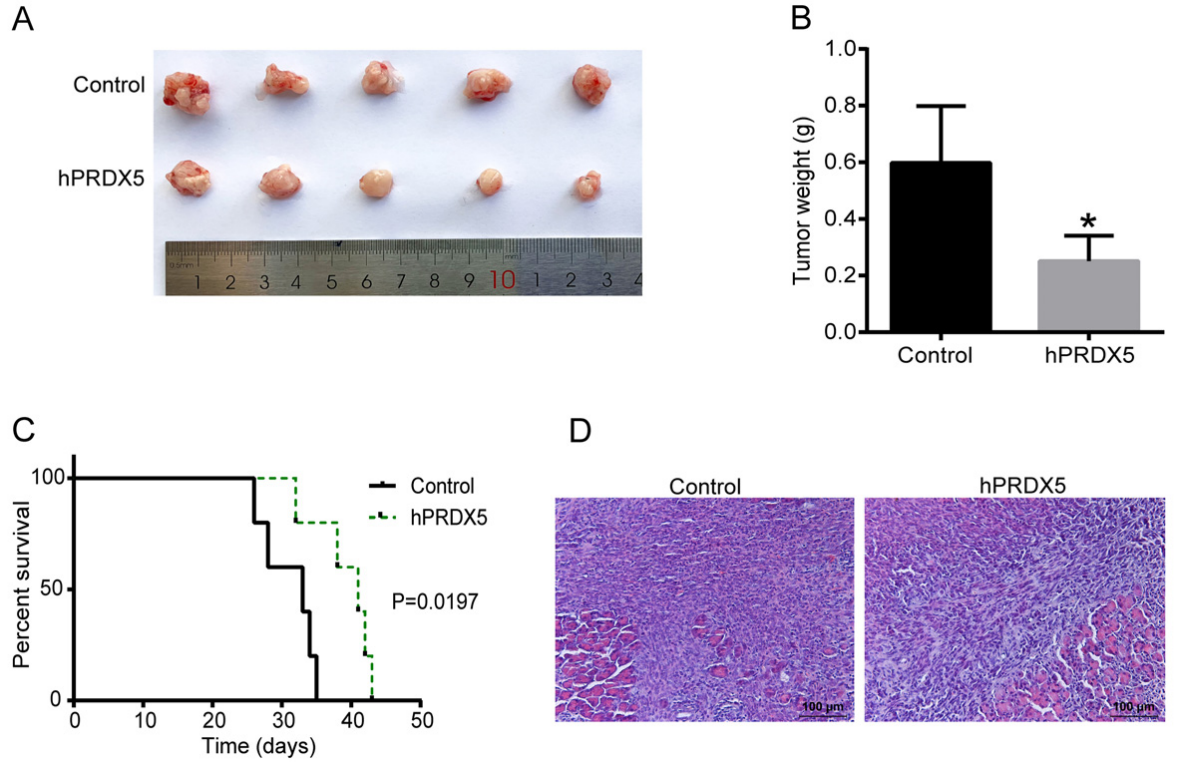
Treatment with human peroxiredoxin-5 (hPRDX5) alone exhibited *in vivo* antitumor activity in a mouse orthotopic transplantation model. **A**, Images of tumor tissues from tumor-bearing C57BL/6 mice. The tumors in the hPRDX5-treated group were smaller than those in the control group. **B**, The tumor weights decreased in hPRDX5-treated mice. **C**, The survival period was prolonged in hPRDX5-treated mice. **D**, Representative images of H&E staining of mouse pancreatic tumor (scale bar 100 μm). Data are reported as means±SD. *P<0.05 compared with the control (*t*-test).

To study the antitumor activity of hPRDX5 *in vivo*, Panc02 cell lines were used for orthotopic pancreas transplantation in mice. Mice were treated with a daily dose of 10 mg/kg of hPRDX5, and the treatment resulted in significant inhibition of tumor weight (Figure 1A and B). The average tumor weight of the hPRDX5 treatment group was 0.25 g, which was significantly lower than that of the control group (mean 0.59 g), and the tumor inhibition rate reached 57.6%. In the hPRDX5 treated group, the inhibition of tumor growth resulted in a significant survival benefit; the survival time of mice was prolonged from 33 to 41 days, and the difference was statistically significant (Figure 1C). Tumor tissues were also paraffin-embedded and were stained with H&E. The histopathological images showed that the vast majority of cells were tumor cells, with a small amount of acinar cells, and infiltrated inflammatory cells (Figure 1D).

### hPRDX5 enhanced immune response in tumor-bearing mice

**Figure 2:**
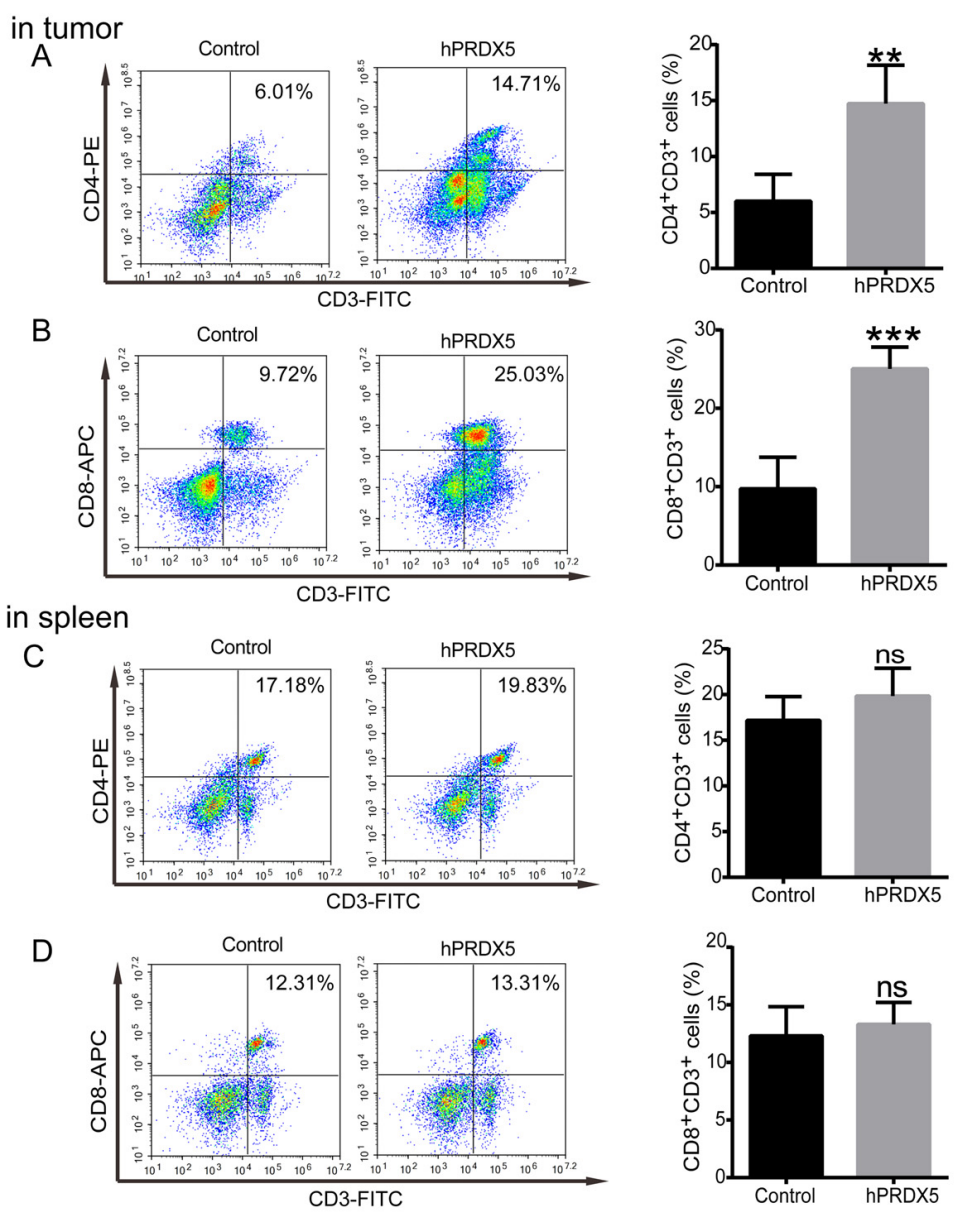
Effect of human peroxiredoxin-5 (hPRDX5) on the proportion of T cells in tumor and spleen tissues of mice. Proportion of CD3+CD4+ T cells (**A**) and CD3+CD8+ T cells (**B**) in tumor tissues and of CD3+CD4+ T cells (**C**) and CD3+CD8+ T cells (**D**) in spleen tissues in the control and hPRDX5 treatment groups. Data are reported as means±SD. **P<0.01 and ***P<0.001 compared with the control (*t*-test). ns: not significant.

**Figure 3:**
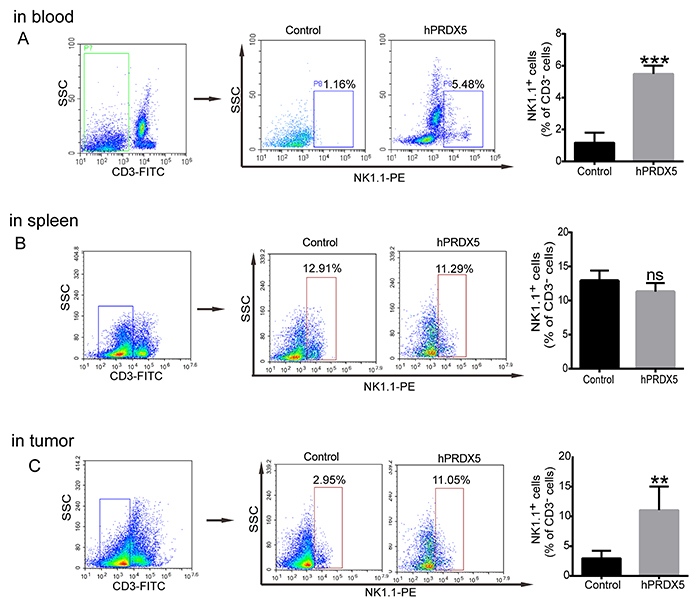
Effect of human peroxiredoxin-5 (hPRDX5) on the proportion of natural killer (NK) cells in peripheral blood (**A**), spleen (**B**), and tumor (**C**) tissues of mice. Data are reported as means±SD. **P<0.01, ***P<0.001 compared with the control (*t*-test). ns: not significant.

**Figure 4:**
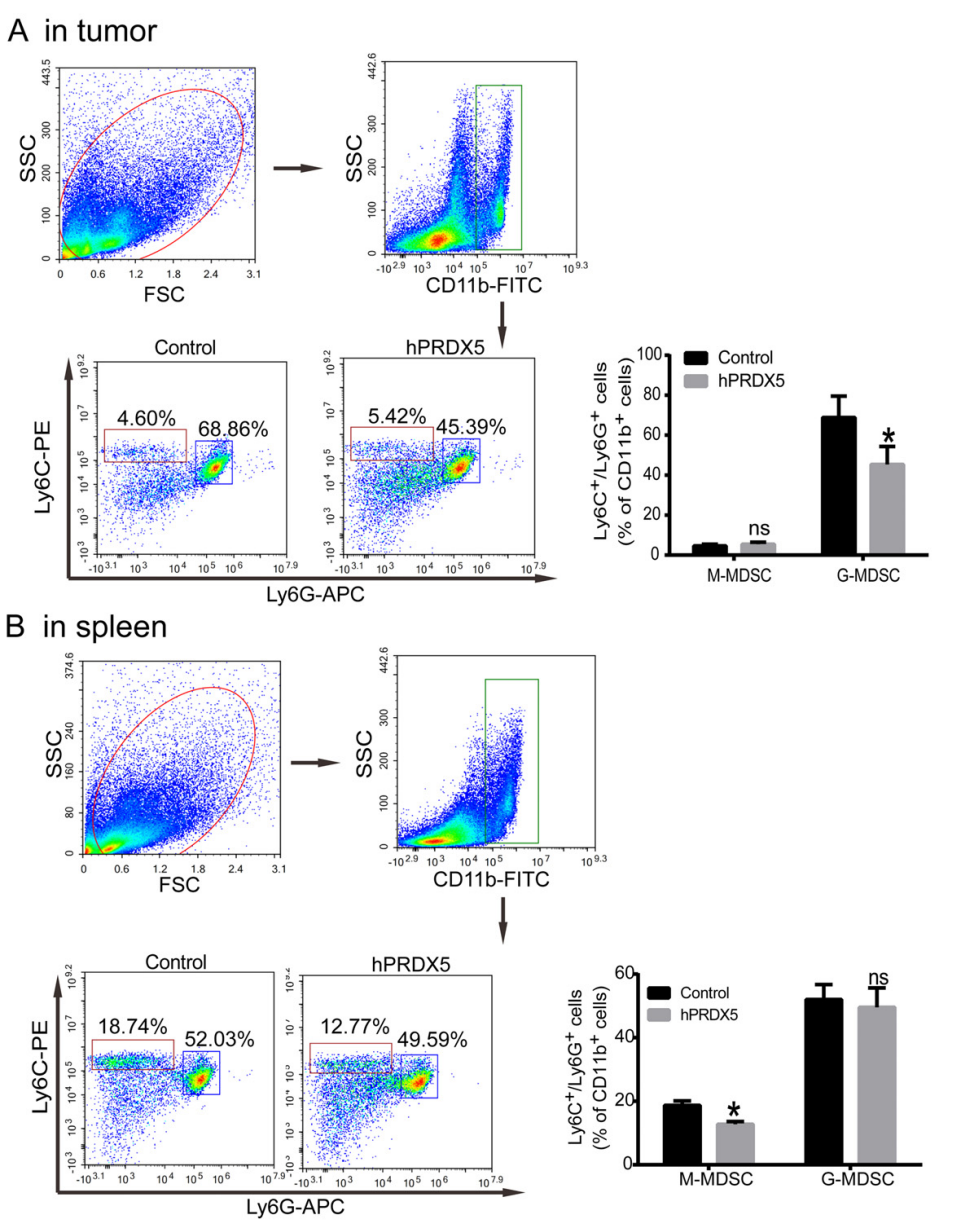
Effect of human peroxiredoxin-5 (hPRDX5) on the proportion of MDSC cells in spleen and tumor tissues of mice. **A**, Proportion of monocytic myeloid-derived suppressor cells (M-MDSC) cells and granulocytic (G)-MDSC cells in tumor tissues (**A**) and in spleen tissues (**B**) in the control group and the hPRDX5 treatment group. Data are reported as means±SD. *P<0.05 compared with the control (*t*-test). ns: not significant.

To explore the mechanism of hPRDX5 in inhibiting the growth of pancreatic cancer, tumor and pancreatic tissues and blood samples were collected to compare the proportion of important immune cells (including CD3+T, CD4+T, CD8+T, MDSC, NK cells, etc.) in the treated and control groups after 14 days of administration. Compared with controls, the infiltration of CD3+CD4+ T cells, CD3+CD8+ T cells, and NK cells was significantly increased by 2-fold, 2-fold, and 4-fold, respectively, in the tumor tissue (Figures 2 and 3). However, G-MDSCs in tumor tissue had a small decrease (Figure 4). All of these data suggested that hPRDX5 can enhance the immune response of important immune cells in the tumor microenvironment. Except for the fact that the proportion of M-MDSCs cells decreased slightly, there was no significant difference in the percentage of T cells, NK cells, and MDSCs between the two groups in the spleen tissue. Specifically, compared with the control group, the proportion of NK cells in the peripheral blood also increased more than 4-fold.

### hPRDX5 enhanced the function of NK cells in tumor tissues

**Figure 5:**
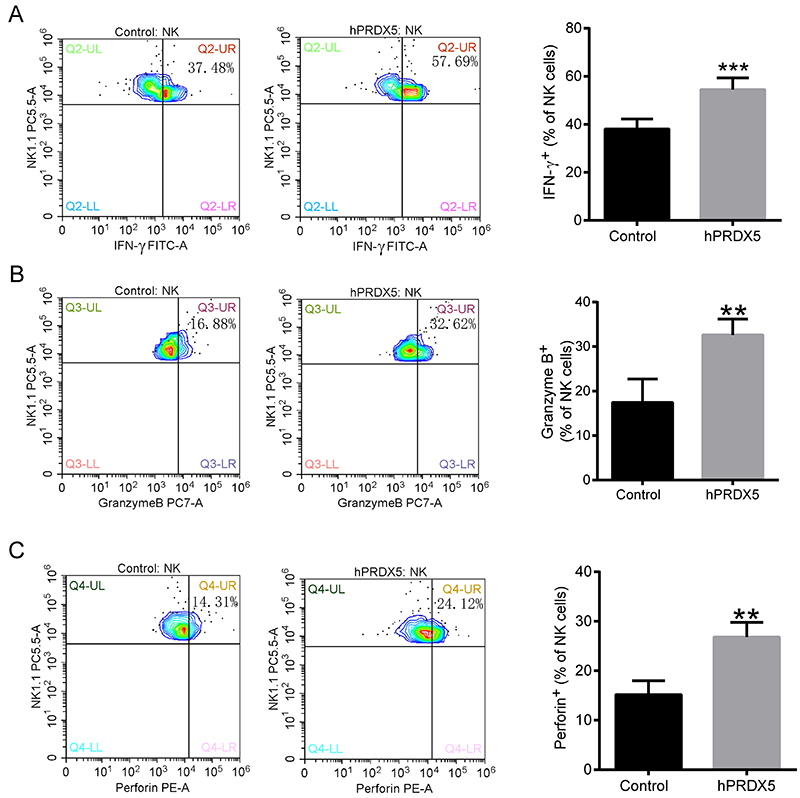
Effect of human peroxiredoxin-5 (hPRDX5) on the expression of function proteins of natural killer (NK) cells in tumor tissues. The levels of IFN-γ (**A**), granzyme B (**B**), and perforin (**C**) in NK cells in the control and hPRDX5 treatment groups. Data are reported as means±SD. **P<0.01 and ***P<0.001 compared with the control (*t*-test).

In view of the remarkable effect of hPRDX5 on the proportion of NK cells in tumor tissues, we decided to further detect the expression of function proteins (such as IFN-γ, granzyme B, and perforin) of NK cells in tumor tissues of tumor-bearing mice in the treatment group and the control group (Figure 5). The results showed that the expression of these three proteins was significantly increased compared with the control group. These cytokines play key roles in promoting the cytotoxicity of NK cells. Therefore, all of these data suggested that hPRDX5 could activate NK cells by both increasing the number of infiltrating NK cells and promoting cytotoxicity.

### hPRDX5 stimulated the proliferation and function of NK cells *in vitro*

**Figure 6:**
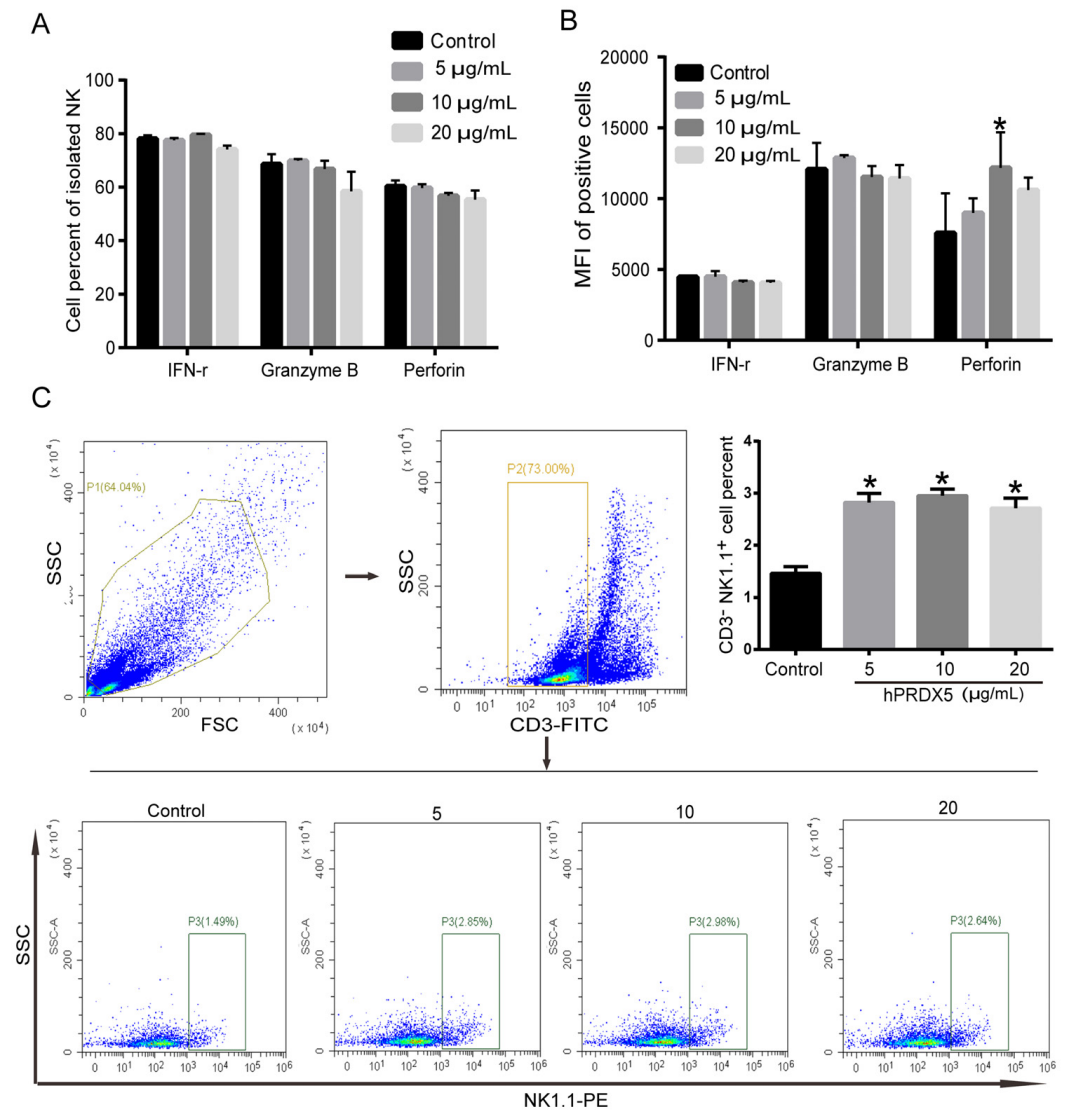
Effect of human peroxiredoxin-5 (hPRDX5) on the expression of function proteins of natural killer (NK) cells *in vitro*. **A**, The levels of IFN-γ, granzyme B, and perforin in NK cells were assessed using flow cytometry. **B**, The mean fluorescent intensity (MFI) of IFN-γ, granzyme B, and perforin in NK cells was measured by flow cytometry. **C**, Proportion of NK cells in bone marrow mononuclear cells stimulated by hPRDX5 for 24 h *in vitro*. Data are reported as means±SD. *P<0.05, compared with the control (ANOVA).

We next examined whether hPRDX5 could activate NK cells *in vitro*. NK cells were isolated from spleen of normal C57BL/6 mice by magnetic bead sorting. After stimulation with hPRDX5 for 24 h *in vitro*, the expression of function proteins in NK cells in the treatment group and the control group was observed. A different result with the *in vivo* experiment was revealed by flow cytometry analysis. There were no significant differences in the levels of function proteins between the two groups (Figure 6A). Only perforin protein expression was slightly augmented by the mean fluorescence intensity analysis (Figure 6B). Bone marrow mononuclear cells were isolated from normal C57BL/6 mice and stimulated by hPRDX5 for 24 h *in vitro*. The experiments showed similar results, as hPRDX5 up-regulated the proportion of NK cells (Figure 6C).

### hPRDX5 activated NK cells through TLR4-MAPK-NF-**κ**B signaling pathway

**Figure 7:**
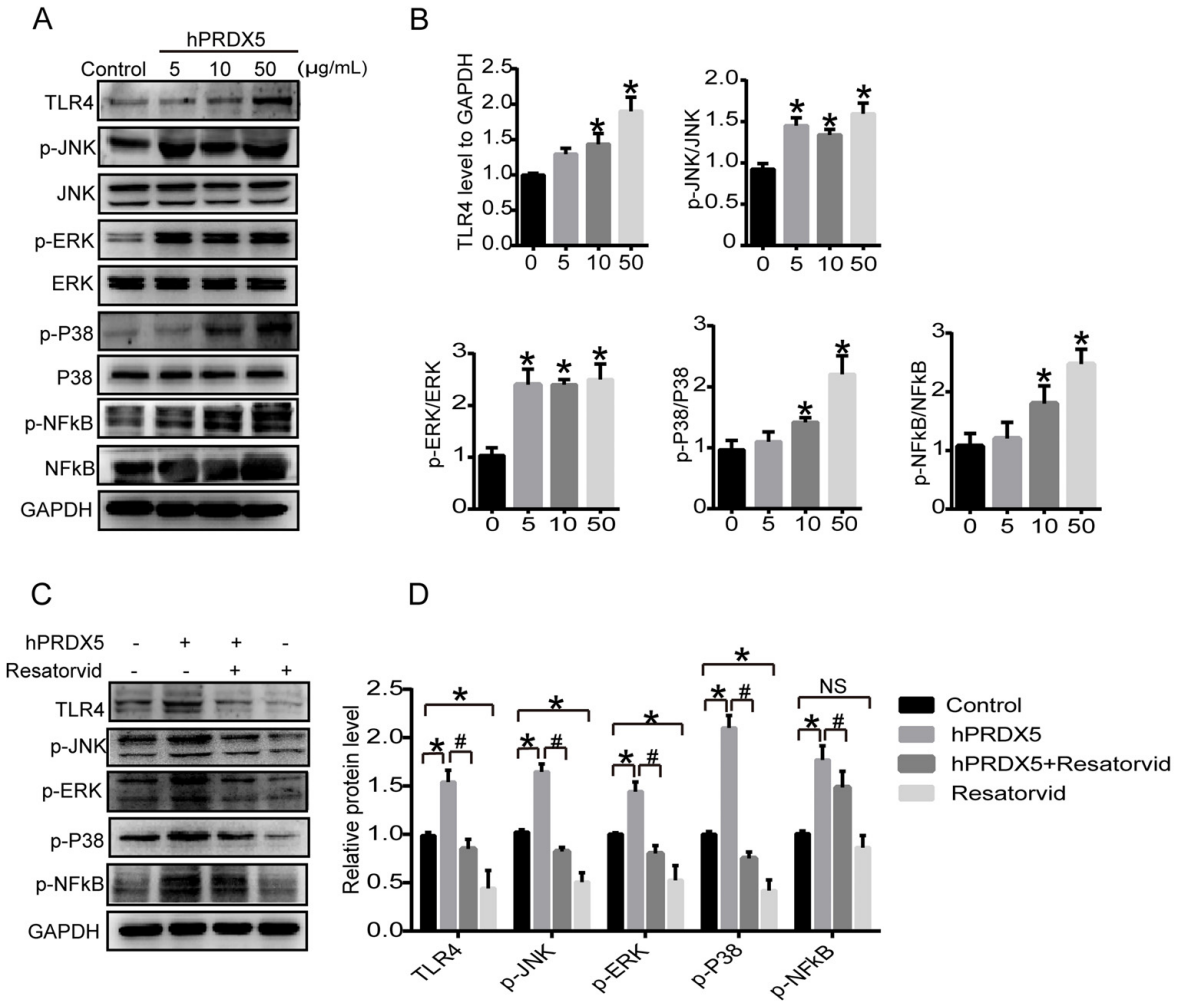
NK92 cells were activated by human peroxiredoxin-5 (hPRDX5) through TLR4-MAPK-NF-κB signaling pathway *in vitro*. **A** and **B**, Levels of downstream protein expressions in NK-92 cells treated with hPRDX5 (5, 10, 50 μg/mL) for 24 h by western blot analysis. **C** and **D**, Levels of downstream protein expressions in NK-92 cells treated with hPRDX5 (50 μg/mL) in the presence or absence of TLR4 inhibitor (resatorvid) by western blotting. Data are reported as means±SD from triplicate wells. *P<0.05, compared with the control. ^#^P<0.05, compared with the relative hPRDX5-treated group (ANOVA). ns: not significant.

Toll-like receptor 4 (TLR4) is one of the key pattern recognition receptors, and it recognizes pathogen-associated molecules and delivers signals to cytoplasm to trigger transcription factors and produce proinflammatory cytokines (18,19). Some compounds exert immunomodulatory biological activities by acting on TLR4 (20). The ability of hPRDX5 to activate NK cells led us to uncover the underlying mechanism. Experimental results showed that hPRDX5 increased the protein level of TLR4 and enhanced the downstream protein expression of p-JNK, p-ERK, p-P38, as well as p-NF-κB. Importantly, TLR4 inhibitor suppressed hPRDX5-induced activation of the TLR4-MAPK-NF-κB signaling pathway, which further proved that hPRDX5 could regulate the function of NK cells by activating TLR4 (Figure 7).

## Discussion

The prominent feature of pancreatic cancers is the immunosuppressive microenvironment. The insufficient ability of anticancer immunity is caused by the recruitment of various types of cells into the tumor, including MDSCs, dendritic cells (DCs), and tumor-infiltrating lymphocytes (TILs) (21). Destroying this immunosuppressive environment in the tumor and promoting the tumor-killing activity of some of the immune cells might provide new options for the treatment of this lethal disease (22).

MDSCs are the population of CD11b+Gr-1+ at early differentiation stages of myeloid cells. They dramatically change during tumor development, infection, and even immunization (23,24). It has been well established that the accumulation of MDSCs suppresses the proliferation and activation of T cells and negatively regulates the maturation of DCs, which together down-regulate the immune responses and cause the tumor cells to escape immune surveillance (24,25). Despite this mode of mechanism, it has been indicated that elimination of the myeloid suppressor cells could reduce tumor-related immunosuppression and improve the efficacy of immunotherapy (26). Therefore, some strategies have been evaluated. Suzuki et al. (27) showed that myeloid suppressor cells that accumulate in animals with large tumors could be selectively eliminated after gemcitabine treatment. The immunosuppressive activity of myeloid suppressor cells on activated NK cells and CD8+ T cells could also be repressed after administration of the chemotherapy drug gemcitabine.

TILs are one of the major components in the tumor microenvironment, including different proportions of CD3+CD4+ (helper T) cells and CD3+CD8+ (cytotoxic T) cells. In patients with pancreatic adenocarcinomas, infiltration of both CD4+ and CD8+ T cells is associated with better prognosis and significantly improved 5-year survival (28-30). In general, accumulated tumor-infiltrating NK cells are associated with a good prognosis in a number of solid tumors. Hence, both NK cells and T cells play an important role in the process of innate and adaptive immunity in clearing up the target tumor cells. In this study, hPRDX5 was shown to have an effect on both tumor-associated lymphocytes and MDSCs. Compared with controls, the infiltration of CD3+CD4+ T cells, CD3+CD8+ T cells, and NK cells was significantly increased in the tumor tissue and the percentage of G-MDSCs decreased to a certain extent. Hence, we summarized that hPRDX5 exerted its anti-tumor activity by changing the populations of immune cells and immunosuppressive cells in the TME.

NK cells are cytotoxic lymphocytes, playing a vital role in immunological surveillance against viral infections and cancer. Unlike the traditional CD8+ T cells, the function of NK cells is triggered and modulated by a balance of inhibitory and activating receptors (20). Moreover, the phenotypes and function of NK cells tend to be strongly regulated by the highly dynamic TME, considering that the functions of NK cells are usually impaired in cancer patients (31). Activated NK cells act on cancer via various pathways mainly through the cytotoxicity pathway and production of cytokines IFN-γ and TNF-α (32). In this study, hPRDX5 was shown to elevate the expression of function proteins of NK cells in tumor tissues. Hence, we concluded that hPRDX5 improved the action of NK cells *in vivo*, and the improved cytotoxicity and quantity of NK played a major role in hPRDX5-induced inhibition against tumor growth *in vivo*.

Toll-like receptors (TLRs) belong to pattern-recognition receptors (PRRs), which are the mammalian homologues of drosophila Toll protein, and play a role in recognition and regulation of the immune response (33,34). TLR4 was the first discovered TLR in humans that could activate the immune-related genes. TLR4 is expressed on antigen presenting cells (APCs), including macrophages and DCs. After stimulation by various ligands, a great number of proinflammatory substances such as cytokines, chemokines, and their receptors are generated through both MyD88-dependent and -independent pathways (35). One of them, TNF-α, which has the ability to trigger DC maturation and migration following proliferation of T helper 1 (Th1) lymphocytes, plays an important role in the TLR4 anticancer effect. On the one hand, DC maturation facilitates cross-presentation of tumor antigens to specific CD8+ T cells (36). On the other hand, the enrichment of IFN-α and IL-12 in TME leads to the recruitment of T cells to the region, whereas some cytokines activate the CD4+ Th1 and consequently CD8+ T cells show antitumor responses, and these are believed to be indispensable for the implementation of antitumor effects (37,38). Hence, we concluded that hPRDX5 could activate TLR4 signaling pathway and result in the activation of NK cells. Moreover, the effect of hPRDX5 on NK cells could be attenuated by the inhibitor of TLR4.

Taken together, the present study provided experimental evidence that recombinant hPRDX5 inhibited pancreatic cancer growth in mice and prolonged the survival of tumor-bearing mice. hPRDX5 was shown to play the anti-tumor effect by regulating the populations of immune cells and immunosuppressive cells in the TME and activate NK cells most likely via the TLR4 signaling pathway.

## References

[1] Rahib L, Smith BD, Aizenberg R, Rosenzweig AB, Fleshman JM, Matrisian LM (2014). Projecting cancer incidence and deaths to 2030: the unexpected burden of thyroid, liver, and pancreas cancers in the United States. Cancer Res.

[2] Siegel R, Ma J, Zou Z, Jemal A (2014). Cancer statistics, 2014. CA Cancer J Clin.

[3] Bellone G, Turletti A, Artusio E, Mareschi K, Carbone A, Tibaudi D (1999). Tumor-associated transforming growth factor-beta and interleukin-10 contribute to a systemic Th2 immune phenotype in pancreatic carcinoma patients. Am J Pathol.

[B04] Park H, Li Z, Yang XO, Chang SH, Nurieva R, Wang YH (2005). A distinct lineage of CD4 T cells regulates tissue inflammation by producing interleukin 17. Nat Immunol.

[B05] Gnerlich JL, Mitchem JB, Weir JS, Sankpal NV, Kashiwagi H, Belt BA (2010). Induction of Th17 cells in the tumor microenvironment improves survival in a murine model of pancreatic cancer. J Immunol.

[6] Yamamoto M, Kamigaki T, Yamashita K, Hori Y, Hasegawa H, Kuroda D (2009). Enhancement of anti-tumor immunity by high levels of Th1 and Th17 with a combination of dendritic cell fusion hybrids and regulatory T cell depletion in pancreatic cancer. Oncol Rep.

[7] Wachsmann MB, Pop LM, Vitetta ES (2012). Pancreatic ductal adenocarcinoma: a review of immunologic aspects. J Investig Med.

[8] Ikemoto T, Yamaguchi T, Morine Y, Imura S, Soejima Y, Fujii M (2006). Clinical roles of increased populations of Foxp3+CD4+ T cells in peripheral blood from advanced pancreatic cancer patients. Pancreas.

[9] Kitayama J, Atomi Y, Nagawa H, Kuroda A, Mutoh T, Minami M (1993). Functional analysis of TCR gamma delta+ T cells in tumour-infiltrating lymphocytes (TIL) of human pancreatic cancer. Clin Exp Immunol.

[10] Degrate L, Nobili C, Franciosi C, Caprotti R, Brivio F, Romano F (2009). Interleukin-2 immunotherapy action on innate immunity cells in peripheral blood and tumoral tissue of pancreatic adenocarcinoma patients. Langenbecks Arch Surg.

[11] Funa K, Nilsson B, Jacobsson G, Alm GV (1984). Decreased natural killer cell activity and interferon production by leucocytes in patients with adenocarcinoma of the pancreas. Br J Cancer.

[12] Aparicio-Pagés MN, Verspaget HW, Peãa AS, Lamers CB (1991). Natural killer cell activity in patients with adenocarcinoma in the upper gastrointestinal tract. J Clin Lab Immunol.

[13] Gabrilovich DI, Nagaraj S (2009). Myeloid-derived suppressor cells as regulators of the immune system. Nat Rev Immunol.

[14] Youn JI, Nagaraj S, Collazo M, Gabrilovich DI (2008). Subsets of myeloid-derived suppressor cells in tumor-bearing mice. J Immunol.

[B15] Condamine T, Gabrilovich DI (2011). Molecular mechanisms regulating myeloid-derived suppressor cell differentiation and function. Trends Immunol.

[16] Movahedi K, Guilliams M, Van den Bossche J, Van den Bergh R, Gysemans C, Beschin A (2008). Identification of discrete tumor-induced myeloid-derived suppressor cell subpopulations with distinct T cell-suppressive activity. Blood.

[17] Feng X, Liu J, Fan S, Liu F, Li Y, Jin Y (2016). The identification of goat peroxiredoxin-5 and the evaluation and enhancement of its stability by nanoparticle formation. Sci Rep.

[18] Zhou L, Liu Z, Wang Z, Yu S, Long T, Zhou X (2017). Astragalus polysaccharides exerts immunomodulatory effects via TLR4-mediated MyD88-dependent signaling pathway *in vitro* and *in vivo*. Sci Rep.

[19] Fang W, Bi D, Zheng R, Cai N, Xu H, Zhou R (2017). Identification and activation of TLR4-mediated signalling pathways by alginate-derived guluronate oligosaccharide in RAW264.7 macrophages. Sci Rep.

[20] Yang Y, Neo SY, Chen Z, Cui W, Chen Y, Guo M (2020). Thioredoxin activity confers resistance against oxidative stress in tumor-infiltrating NK cells. J Clin Invest.

[21] Wörmann SM, Diakopoulos KN, Lesina M, Algül H (2014). The immune network in pancreatic cancer development and progression. Oncogene.

[22] Garrido-Laguna I, Hidalgo M (2015). Pancreatic cancer: from state-of-the-art treatments to promising novel therapies. Nat Rev Clin Oncol.

[23] Serafini P, De Santo C, Marigo I, Cingarlini S, Dolcetti L, Gallina G (2004). Derangement of immune responses by myeloid suppressor cells. Cancer Immunol Immunother.

[24] Gallina G, Dolcetti L, Serafini P, De Santo C, Marigo I, Colombo MP (2006). Tumors induce a subset of inflammatory monocytes with immunosuppressive activity on CD8+ T cells. J Clin Invest.

[25] Frey AB (2006). Myeloid suppressor cells regulate the adaptive immune response to cancer. J Clin Invest.

[26] Bronte V, Serafini P, Apolloni E, Zanovello P (2001). Tumor-induced immune dysfunctions caused by myeloid suppressor cells. J Immunother.

[27] Suzuki E, Kapoor V, Jassar AS, Kaiser LR, Albelda SM (2005). Gemcitabine selectively eliminates splenic Gr-1+/CD11b+ myeloid suppressor cells in tumor-bearing animals and enhances antitumor immune activity. Clin Cancer Res.

[28] Ino Y, Yamazaki-Itoh R, Shimada K, Iwasaki M, Kosuge T, Kanai Y (2013). Immune cell infiltration as an indicator of the immune microenvironment of pancreatic cancer. Br J Cancer.

[B29] Fukunaga A, Miyamoto M, Cho Y, Murakami S, Kawarada Y, Oshikiri T (2004). CD8+ tumor-infiltrating lymphocytes together with CD4+ tumor-infiltrating lymphocytes and dendritic cells improve the prognosis of patients with pancreatic adenocarcinoma. Pancreas.

[30] Sideras K, Braat H, Kwekkeboom J, van Eijck CH, Peppelenbosch MP, Sleijfer S (2014). Role of the immune system in pancreatic cancer progression and immune modulating treatment strategies. Cancer Treat Rev.

[31] Smyth MJ, Hayakawa Y, Takeda K, Yagita H (2002). New aspects of natural-killer-cell surveillance and therapy of cancer. Nat Rev Cancer.

[32] Hodge G, Barnawi J, Jurisevic C, Moffat D, Holmes M, Reynolds PN (2014). Lung cancer is associated with decreased expression of perforin, granzyme B and interferon (IFN)-gamma by infiltrating lung tissue T cells, natural killer (NK) T-like and NK cells. Clin Exp Immunol.

[33] Adams S (2009). Toll-like receptor agonists in cancer therapy. Immunotherapy.

[34] Drexler SK, Foxwell BM (2010). The role of toll-like receptors in chronic inflammation. Int J Biochem Cell Biol.

[35] Uto T, Akagi T, Yoshinaga K, Toyama M, Akashi M, Baba M (2011). The induction of innate and adaptive immunity by biodegradable poly(gamma-glutamic acid) nanoparticles via a TLR4 and MyD88 signaling pathway. Biomaterials.

[36] Balkwill F (2009). Tumour necrosis factor and cancer. Nat Rev Cancer.

[37] Oblak A, Jerala R (2011). Toll-like receptor 4 activation in cancer progression and therapy. Clin Dev Immunol.

[38] Stier S, Maletzki C, Klier U, Linnebacher M (2013). Combinations of TLR ligands: a promising approach in cancer immunotherapy. Clin Dev Immunol.

